# P-290. PrEP-aring Internal Medicine Residents for HIV Pre-Exposure Prophylaxis (PrEP): A Quality Improvement Intervention in a Community Hospital

**DOI:** 10.1093/ofid/ofaf695.511

**Published:** 2026-01-11

**Authors:** Aygul Iskandarova, Alay Nanavati, Dimitra Skiada, Samira Reyes Dassum

**Affiliations:** Roger Williams Medical Center, Providence, RI; Roger Williams Medical Center, Providence, RI; Chartercare Medical Associates, PROVIDENCE, Rhode Island; Roger Williams Medical Center, Providence, RI

## Abstract

**Background:**

PrEP is vital for HIV prevention, yet few residents receive training, revealing a learning gap. We aimed to improve PrEP knowledge and prescribing comfort among internal medicine residents.

**Figure 1.**
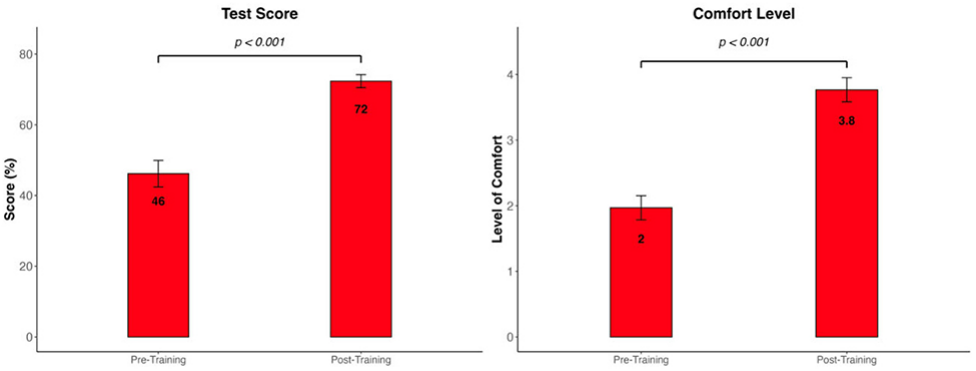
Changes in medical knowledge and level of comfort in prescribing HIV PrEP before and after intervention

**Figure 2.**
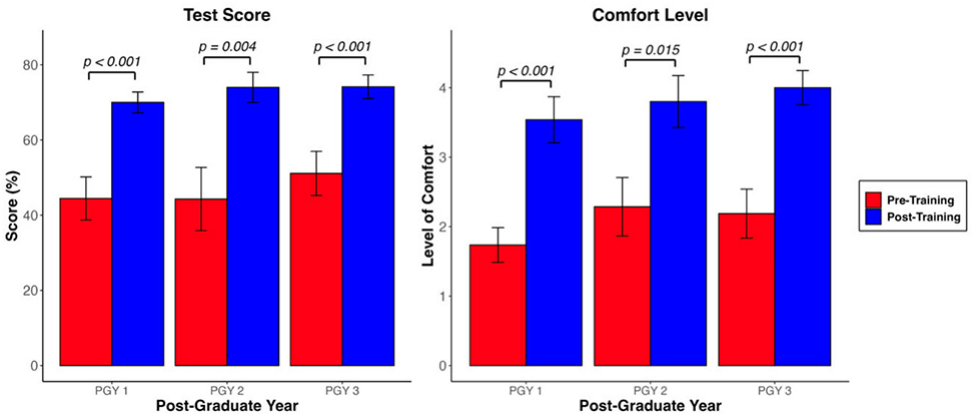
Changes in HIV PrEP knowledge assessment and level of comfort before and after intervention by post-graduate year

**Methods:**

In this quasi-experimental study, residents completed a 10-question multiple-choice test before and after the intervention, reported their post graduate year (PGY), their comfort prescribing PrEP (1–5 Likert scale), and described challenges in free-text responses, which were categorized into themes: sexual history-taking, PrEP indications, medication options and side effects, counseling, and cost.

From 2/2025-4/2025, residents attended PrEP focused didactic sessions, including a simulated case with feedback. Supervising clinicians received a separate lecture.

Ranked and standard linear models evaluated changes in test scores and comfort levels (p < 0.05), with data analyzed using R (v4.1.2).

**Figure 3.**
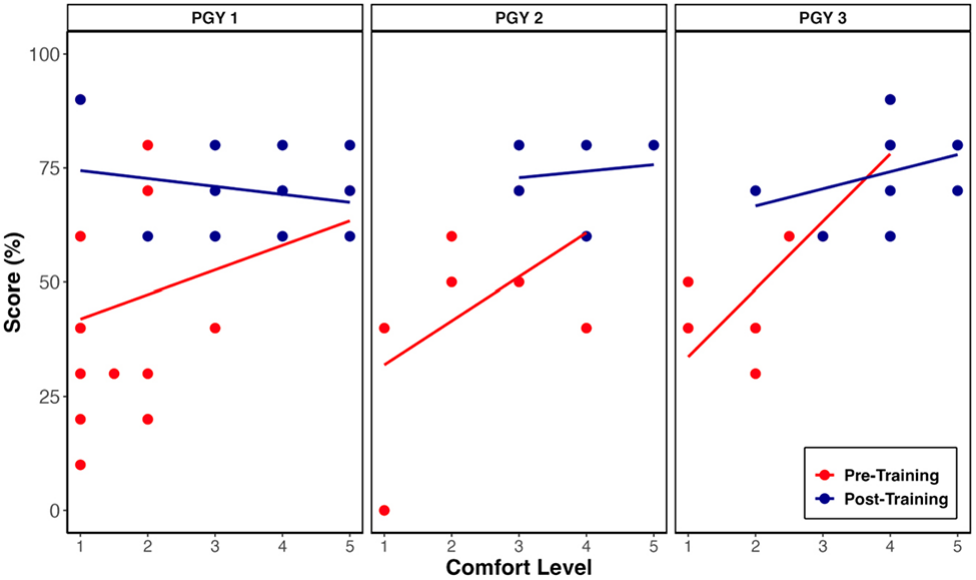
Change in medical knowledge and level of comfort in prescribing HIV PrEP before and after intervention by post-graduate year

**Table 1.**
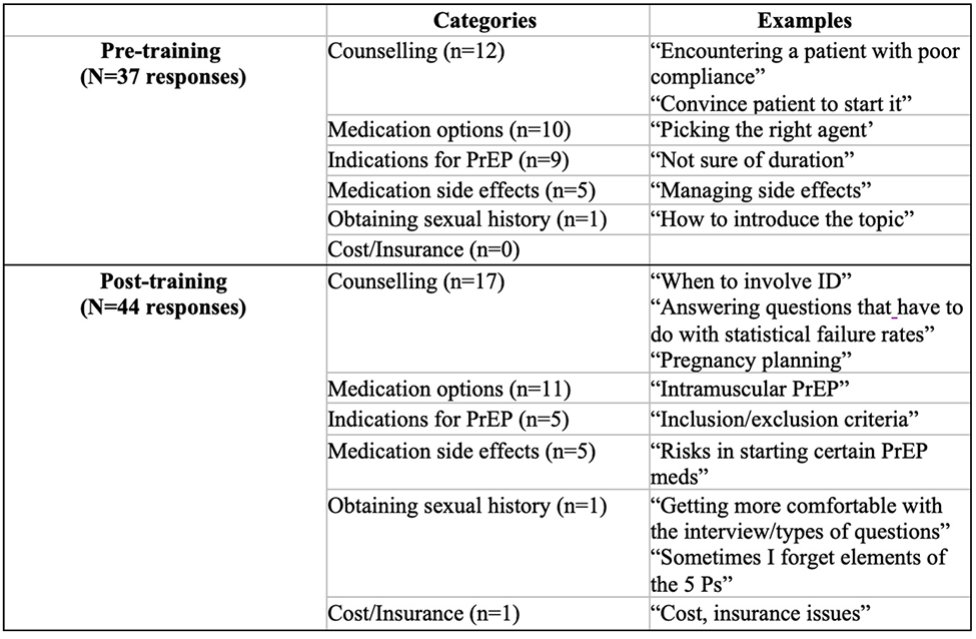
Resident reported challenges in prescribing HIV PrEP

**Results:**

Of 46 eligible residents, 34 (74%) completed pre-training test and 30 (65%) residents completed the post-training test. Overall test scores improved by 26% (p< 0.001). Comfort level improved by 1.8 points (p< 0.001). (Figure 1). Test scores and comfort levels improved across all PGY (Figure 2).

Higher test scores correlated with higher confidence among all PGY levels. (Figure 3)

Reported challenges were grouped and quantified as shown in Table 1. The most reported challenges were counselling (n =12) and medication options (n = 10) despite training.

**Conclusion:**

Our targeted intervention significantly improved PrEP knowledge and comfort, aligning with prior studies, though few assessed practical skills.

Post-training, residents felt more confident identifying PrEP candidates, but challenges with counseling and medication selection persisted, suggesting these need continued support. Some residents with better scores still reported low comfort, pointing to non-cognitive barriers like personal discomfort.

Limitations include a small sample size and no assessment of clinical outcomes like prescribing rates.

This intervention effectively enhanced resident knowledge and confidence and can be integrated into residency training to support HIV prevention.

**Disclosures:**

All Authors: No reported disclosures

